# Supplementary material for: Fecal microbiota transplantation mitigates respiratory infection in rats exposed to hypobaric hypoxia by modulating the NLRP3 inflammasome and mucosal immunity
Source: PLoS One. 2026 Apr 28;21(4):e0347857. doi: 10.1371/journal.pone.0347857 (PMC13124060; doi:10.1371/journal.pone.0347857)

information

Exposure instrument: Azure 300
Exposure time: 30 s

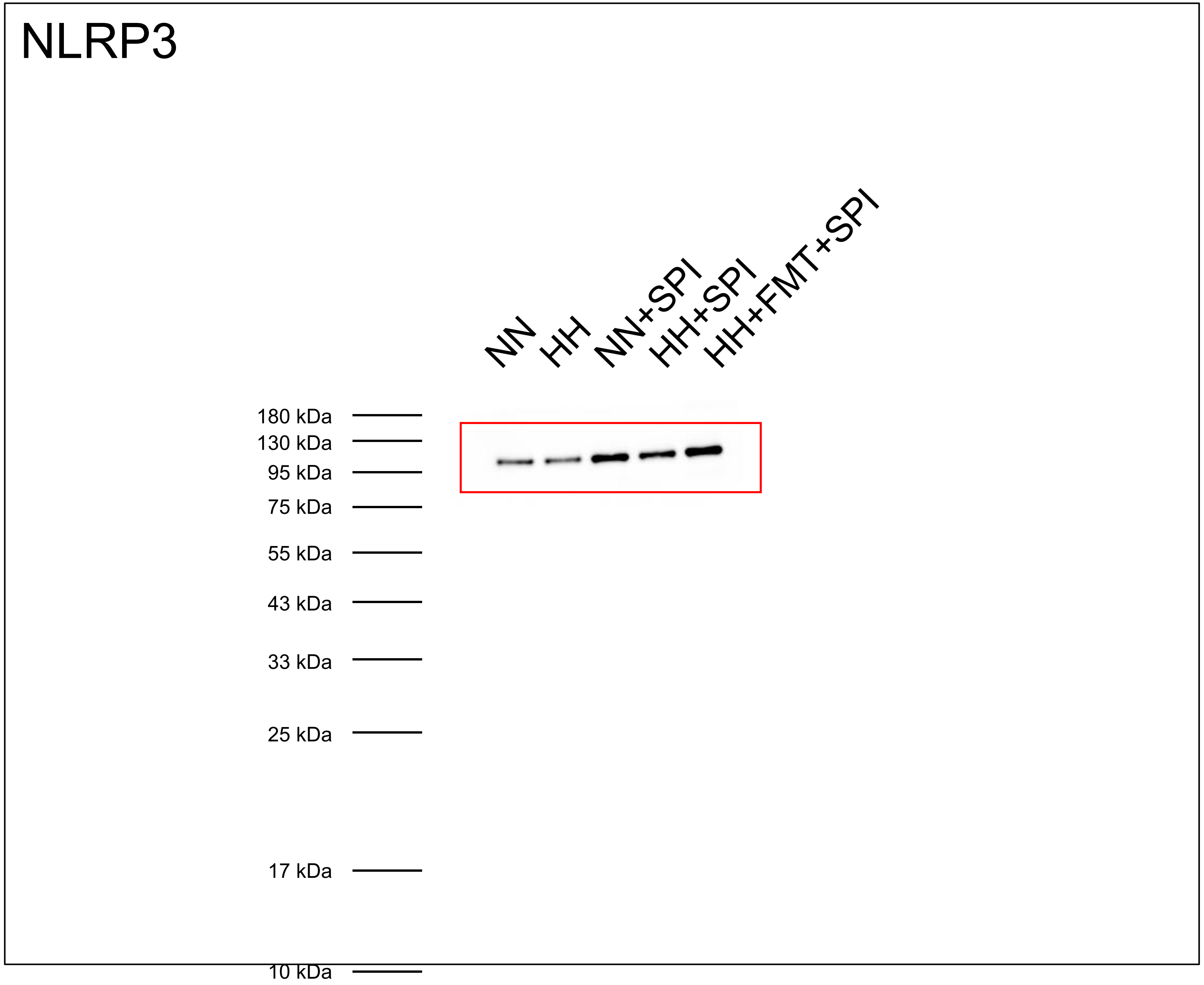

information

Exposure instrument: Azure 300
Exposure time: 30 s

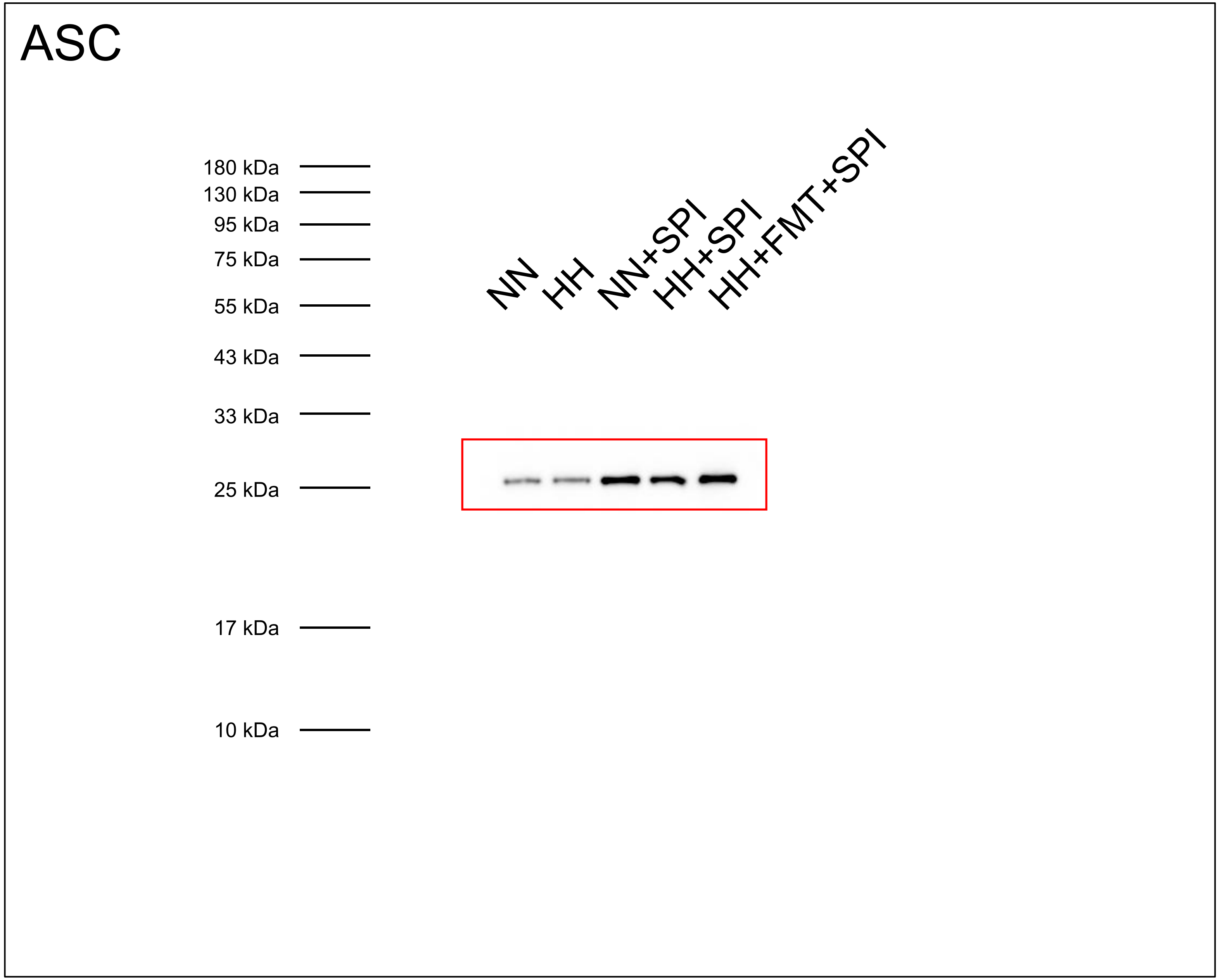

information

Exposure instrument: Azure 300
Exposure time: 30 s

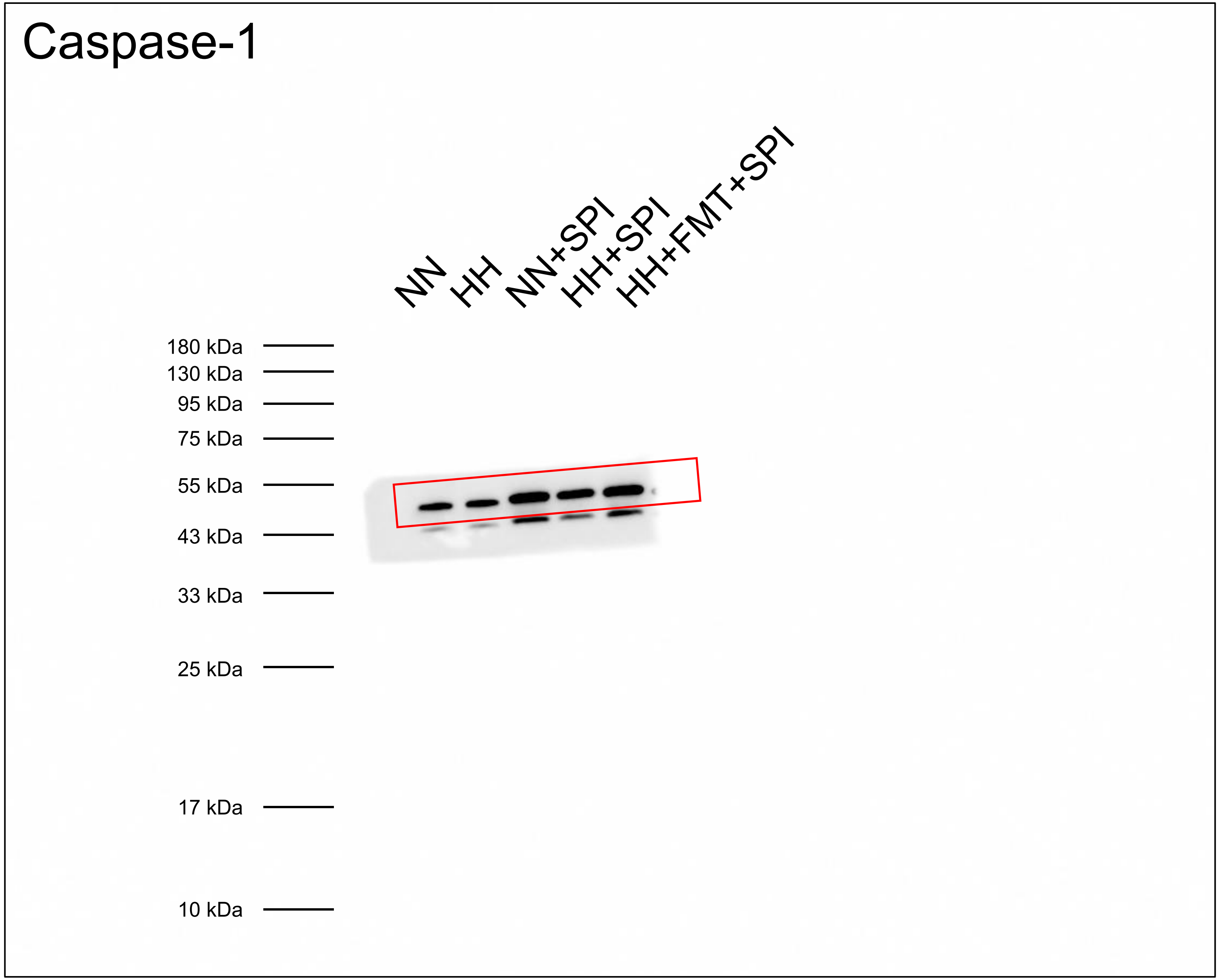

information

Exposure instrument: Azure 300
Exposure time: 5 s

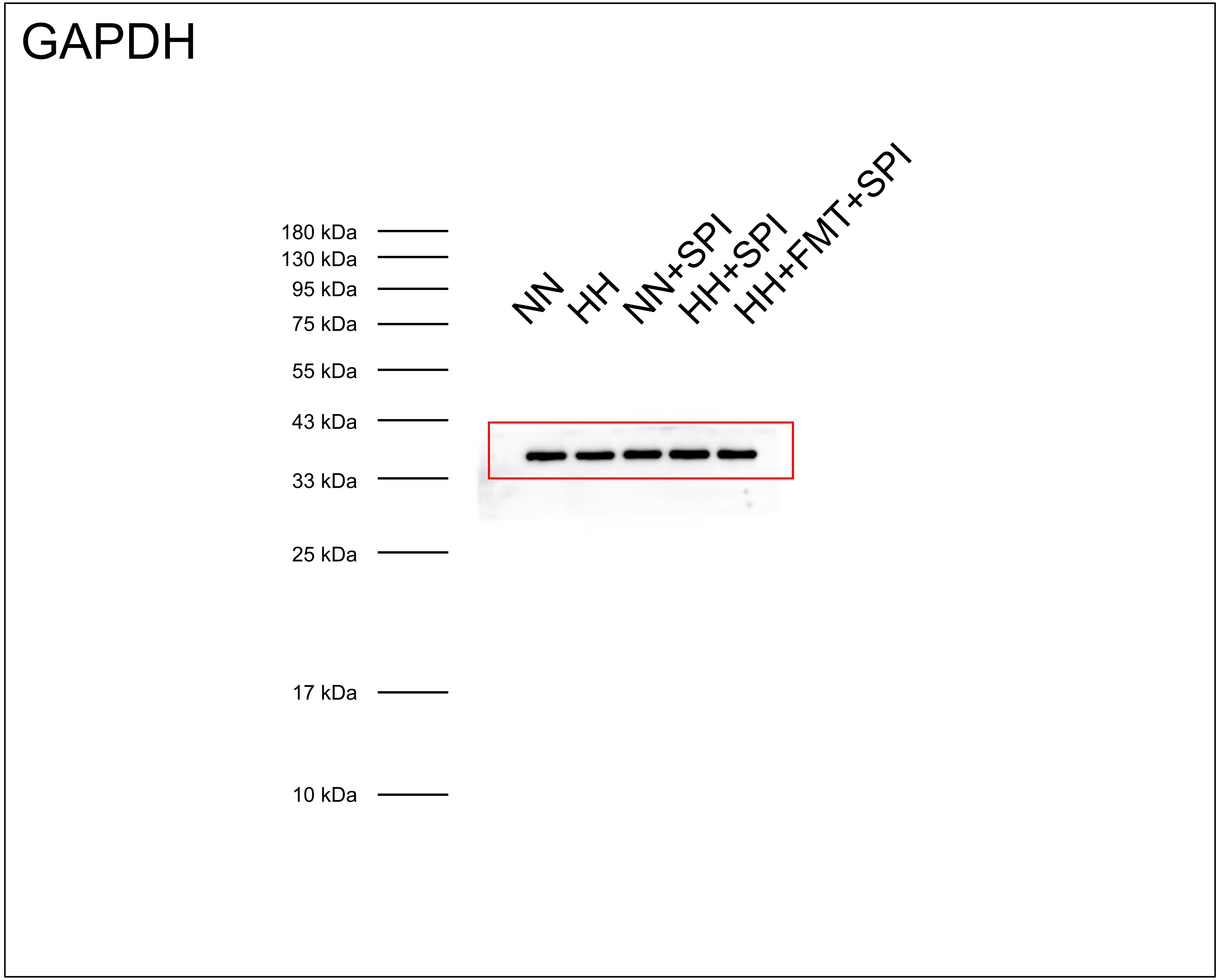

Supplement: S1 Fig — Effects of FMT on NLRP3, ASC and Caspase-1 protein expressions in the airway mucosal tissues of rats. (PDF) [file pone.0347857.s001.pdf]
