# Supplementary material for: Fecal microbiota transplantation mitigates respiratory infection in rats exposed to hypobaric hypoxia by modulating the NLRP3 inflammasome and mucosal immunity
Source: PLoS One. 2026 Apr 28;21(4):e0347857. doi: 10.1371/journal.pone.0347857 (PMC13124060; doi:10.1371/journal.pone.0347857)
Supplement: Attachment — Submitted filename: Response to Reviewers.docx [file pone.0347857.s003.docx]

Response to Reviewers' Comments

We sincerely thank all the reviewers for their careful evaluation and constructive feedback on our manuscript. We have carefully considered each comment and have revised the manuscript accordingly. Our detailed point-by-point responses are provided below.

Reviewer #1: (1)Line 135~137, “However, the gut microbiota's effect on NLRP3 inflammasome activity and airway mucosal immune barrier function under respiratory infection induced by high altitude exposure is unclear” Regarding this issue, the results and discussion section in the manuscript still failed to provide a clear expression.

*Response:We thank the reviewer for this important observation. We have now strengthened the discussion to explicitly link our findings to the question raised in the introduction. See lines 410-450 in the revised manuscript.Gut microbiota transplantation can to some extent restore NLRP3 dysfunction and damage to the respiratory mucosal immune barrier caused by hypoxia.*

(2) There are many grammatical and formatting errors in this manuscript.

*Response:We deeply apologize for these oversights. The manuscript has been thoroughly proofread and edited for language again to correct grammatical errors, improve sentence fluency, and ensure consistent formatting (e.g., italicization of genus/species names, uniform reference formatting, and standardized presentation of figures and tables). It has also been revised by a professional language polishing company，the current version has been significantly improved in terms of language quality and presentation.*

(3) Line362~368, “The protein expressions of NLRP3, ASC and Caspase-1 in the airway epithelial tissue of the HH + SPI group were increased after being subjected to streptococcus pneumoniae infection for 3 days after hypobaric hypoxia challenge (P < 0.05, compared to the NN group). However, the degree of change in NLRP3, ASC and Caspase-1 protein expression of the HH + SPI group were significantly lower compared to the NN + SPI group (P < 0.05) (Figure 3A; Figure 3B). After FMT, the protein expression levels of NLRP3, ASC, and Caspase-1 in the airway mucosal tissue of rats in the HH + SPI + FMT group were restored (P < 0.05, compared to the NN + SPI group)”. Line 370~373, “The levels of IL-1β, TNF-α, and IL-6 in the BALF of the HH + SPI group subjected to streptococcus pneumoniae infection were decreased (P<0.05, compared with the NN + SPI group). After FMT, the levels of IL-1β, TNF-α, and IL-6 of the HH + FMT + SPI group subjected to streptococcus pneumoniae infection were restored”.

In general, pneumococcal infection leads to the activation of various signaling pathways including NLRP3 inflammasome, resulting in the extensive expression of pro-inflammatory factors, which triggers a cytokine storm and causes lung damage. However, the results of this manuscript were exactly the opposite. Why?

*Response:Thank you very much for your question. In respiratory bacterial infections, the activation of NLRP3 is a 'double-edged sword'. On one hand, it promotes the maturation and release of pro-inflammatory cytokines such as IL-1β and IL-18, which can recruit and activate immune cells like neutrophils and macrophages, thereby enhancing the body's ability to clear bacteria. On the other hand, when the infection is too severe or the body's ability to clear bacteria is insufficient, excessive activation of NLRP3 can lead to uncontrolled inflammatory responses, triggering a 'cytokine storm'. In our low-pressure hypoxic rat airways, we observed that normal activation of NLRP3 was inhibited, while fecal microbiota transplantation partially restored NLRP3 function without causing excessive activation.We have added to the discussion section regarding Potential Explanation for the Seemingly Paradoxical Immune Modulation under Hypobaric Hypoxia,Detailed information can be found in the discussion section.*

*We have also optimized the language in the results section you mentioned to make the comparison clearer.section 3.4 of the Results part for details*

Reviewer #3: I appreciate the authors' revisions. However, I must point out a persisting issue with Figure 4. Columbia Blood Agar plates are typically red, but the plates shown in the Figure 4 do not appear to be blood agar plates. The authors should verify if the correct images were uploaded or clarify the discrepancy between the methods described and the figures provided.

*Response:Thank you very much for pointing out this important error. Due to our oversight, the original figure incorrectly used the total colony count data of all bacterial cultures from bronchoalveolar lavage fluid. and replaced it in the main text with a specific colony count figure for Streptococcus pneumoniae culture. We sincerely apologize for any inconvenience caused and deeply appreciate your careful review and valuable comments.*

Reviewer #4: The authors have addressed all the reviewers' comments. This revised version of the manuscript may be accepted for publication.

*Response:We are grateful to Reviewer #4 for their positive assessment and support for publication. We have incorporated all suggestions from the previous and current round of review to strengthen the manuscript.*
